# Supplementary material for: Isolation limits spring pollination in a UK fragmented landscape
Source: PLoS One. 2024 Sep 19;19(9):e0310679. doi: 10.1371/journal.pone.0310679 (PMC11412521; doi:10.1371/journal.pone.0310679)
Supplement: S1 File — (DOCX) [file pone.0310679.s001.docx]

**Supplementary material from Isolation limits spring pollination in a UK fragmented landscape**

***Contents***

- 1. *The landscape*
  2. *Plant survey methods*
  3. *A list of plants identified*

*1.The landscape*


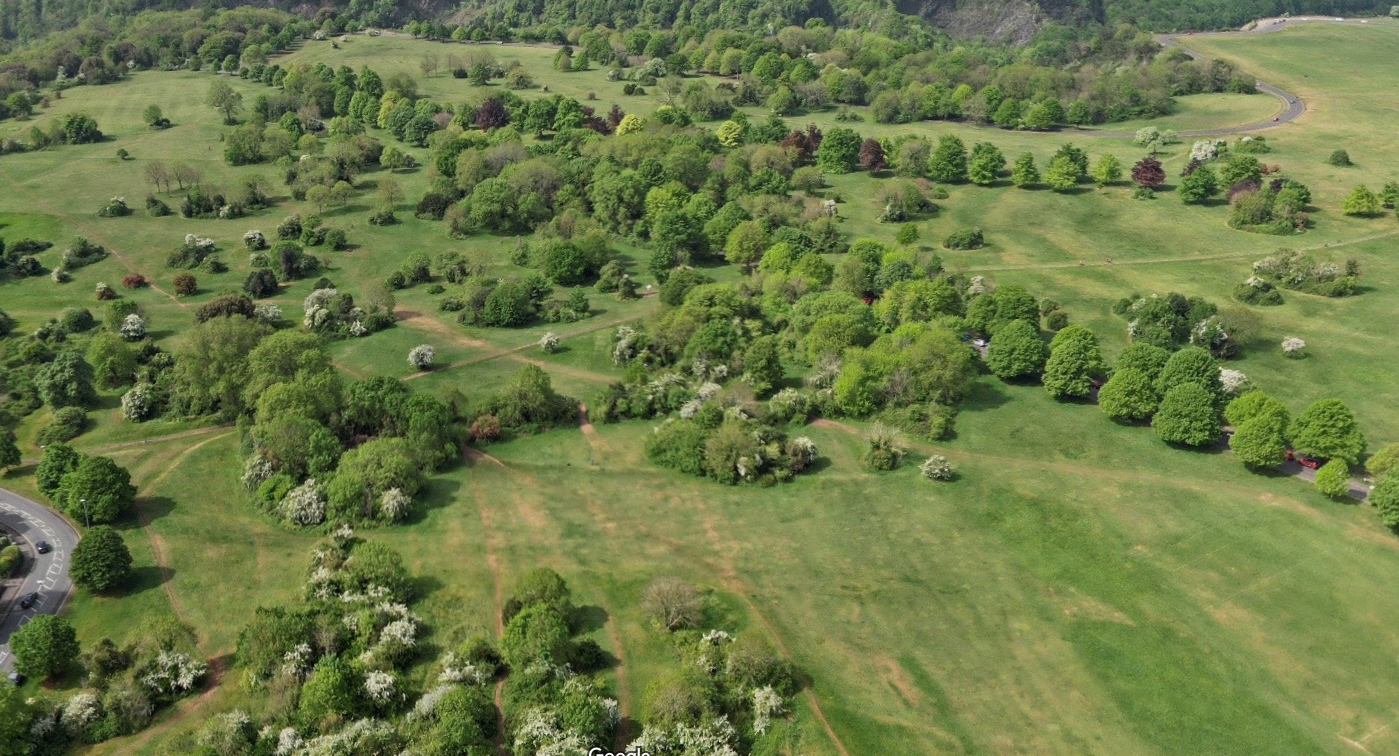


**Figure S1**. Aerial photography of part of the fragmented landscape (provided by Felix Surplus under CC BY 4.0 license). The landscape (< 1.5km^2^) is located at Durdham Downs in the centre of Bristol.

*2. Plant survey methods*

A field survey of plants in the selected patches was conducted in late March 2022, where bumblebee queens started to forage. The survey was done by walking through each woodland patch, recording all the plants to the species level, noting whether they were flowering or not. Any patches with wild bluebells were recorded (n=10).

*3. A list of plant species*

**Table S1** A list of plant species identified by plant survey in the selected 51 patches.

| **Species** | **Common name** | **Flowering** |
| --- | --- | --- |
| *Acer campestre* | Field maple | Yes |
| *Acer negundo* | Boxelder mapple | No |
| *Acer platanoides* | Norway maple | Yes |
| *Acer pseudoplatanus* | Sycamore | No |
| *Achillea millefolium* | Yarrow | No |
| *Aconitum lamarckii* | Northern wolf's-bane | No |
| *Aesculus hippocastanum* | Horse chestnut | No |
| *Alliaria petiolata* | Garlic mustard | Yes |
| *Allium schoenoprasum* | Chives | No |
| *Allium spp.* | Onion spp. | No |
| *Anthriscus spp.* | Carrot family | No |
| *Anthriscus sylvestris* | Cow parsley | Yes |
| *Artemisia vulgaris* | Common mugwort | No |
| *Arum maculatum* | Cuckoo-pint | Yes |
| *Asparagus spp.* | Asparagus | No |
| *Asplenium scolopendrium* | Harts tongue fern | No |
| *Athyrium filix-femina* | Lady fern | No |
| *Bellis perennis* | Common daisy | Yes |
| *Betula pendula* | Silver birch | No |
| *Betula pubescens* | Downy birch | No |
| *Cardamine pratensis* | Cuckooflower | Yes |
| *Carduus acanthoides* | Plumeless thistle | No |
| *Carduus spp.* | Thistle | No |
| *Carpinus betulus* | European hornbeam | No |
| *Cerastium fontanum* | Little mouse-ear chickweed | Yes |
| *Cirsium arvense* | Creeping thistle | No |
| *Cirsium vulgare* | Spear thistle | No |
| *Clematis spp* | *Clematis* | Yes |
| *Clematis vitaba* | Old man's beard | No |
| *Cornus sanguinea* | Common dogwood | No |
| *Crataegus monogyna* | Hawthorn | Yes |
| *Daucus carota* | Wild carrot | No |
| *Dryopteris filix-mas* | Male fern | No |
| *Dryopteris spp.* | Wood fern | Yes |
| *Epilobium spp* | Willowherbs | No |
| *Erigeron strigosus* | Daisy fleabane | Yes |
| *Erodium cicutarium* | Storksbill | Yes |
| *Fagus spp.* | Beech | Yes |
| *Fagus sylvatica* | European beech | No |
| *Ficaria verna* | Lesser Celandine | Yes |
| *Fraxinus excelsior* | European ash | No |
| *Galium aparine* | Catchweed bedstraw | Yes |
| *Galium mollugo* | Wild madder | No |
| *Galium spp* | Cleavers | No |
| *Geranium dissectum* | Cut-leaved crane's-bill | No |
| *Geranium molle* | Dove's-foot crane's-bill | Yes |
| *Geranium robertianum* | Herb Robert | Yes |
| *Geum urbanum* | Wood avens | Yes |
| *Glechoma hederacea* | Ground-ivy | Yes |
| *Hedera canariensis* | Canary Islands Ivy | No |
| *Hedera helix* | Ivy | No |
| *Heracleum sphondylium* | Hogweed | Yes |
| *Hyacinthoides non-scripta* | Bluebell | Yes |
| *Hypochaeris radicata* | Flatweed | No |
| *Ilex aquifolium* | European holly | No |
| *Ilex decidua* | Possumhaw | No |
| *Iris spp.* | Iris | No |
| *Juglans regia* | Persian walnut | No |
| *Lamium album* | White deadnettle | Yes |
| *Ligustrum vulgare* | Common privet | No |
| *Lunaria annua* | Annual honesty | Yes |
| *Malus spp* | Apple | Yes |
| *Mercurialis perennis* | Dog's mercury | No |
| *Myosotis sylvatica* | Forget-me-not | Yes |
| *Persicaria 'vacciniifolia'* | Knotweed | No |
| *Plantago lanceolata* | Ribwort plantain | Yes |
| *Plantago media* | Hoary plantain | No |
| *Plantago spp.* | Plantago | No |
| *Poa annua* | Annual meadow grass | Yes |
| *Primula veris* | Cowslip | No |
| *Prunus avium* | Wild Cherry | Yes |
| *Prunus laurocerasus* | Cherry laurel | Yes |
| *Prunus spinosa* | Blackthorn | No |
| *Pyrus communis* | European pear | Yes |
| *Quercus ilex* | Holm oak | No |
| *Quercus robur* | Pedunculate Oak | No |
| *Quercus spp.* | Oak | No |
| *Ranunculus bulbosus* | Bulbous buttercup | Yes |
| *Ranunculus repens* | Creeping buttercup | Yes |
| *Rosa canina* | Dog rose | No |
| *Rose multiflora* | Multiflora rose | No |
| *Rose spp.* | Rose | Yes |
| *Rubia peregrina* | Wild Madder | No |
| *Rubus fruticosus* | Bramble | No |
| *Rumex obtusifolius* | Broad-leaved dock | No |
| *Sambucus nigra* | Elder | Yes |
| *Sanguisorba minor* | Salad burnet | No |
| *Sisymbrium officinale* | Hedge mustard | Yes |
| *Skimmia japonica* | Japanesese skimmia | Yes |
| *Smyrnium olusatrum* | Alexanders | Yes |
| *Solanum nigrum* | Black night shade | No |
| *Sonchus oleraceus* | Common sow-thistle | No |
| *Stachys sylvatica.* | Hedge woundwork | No |
| *Taraxacum officinale* | Dandelion | Yes |
| *Taxus baccata* | English Yew | No |
| *Tilia cordata* | Lime | No |
| *Tilia platyphyllos* | Large-leaved lime | No |
| *Trifolium repens* | White clover | No |
| *Ulmus glabra* | Wych Elm | No |
| *Ulmus laevis* | European elm | No |
| *Ulmus minor* | Field Elm | No |
| *Ulmus procera* | Common elm | No |
| *Umbellularia californica* | Bay laurel | No |
| *Uritica dioica* | Common nettle | No |
| *Viola odorata* | Common violet | Yes |
| *Viola riviniana* | Common dog violet | Yes |
